# Alum increases antigen uptake, reduces antigen degradation and sustains antigen presentation by DCs *in vitro*

**DOI:** 10.1016/j.imlet.2012.06.002

**Published:** 2012-09

**Authors:** Tirth R. Ghimire, Robert A. Benson, Paul Garside, James M. Brewer

**Affiliations:** a Strathclyde Institute of Pharmacy and Biomedical Sciences, University of Strathclyde, Glasgow, Scotland, United Kingdom; b Institute of Infection, Immunity and Inflammation, 120 Glasgow Biomedical Research Centre, University of Glasgow, Glasgow G12 8TA, Scotland, United Kingdom

**Keywords:** BMDC, bone marrow-derived dendritic cell, EαGFP, Ealpha green fluorescence protein, Alum, Antigen uptake, Processing, Presentation, YAe system

## Abstract

Aluminium adjuvants (alum) have been the only widely approved adjuvants for use in human vaccines since the 1920s, however, the mechanism of action of these adjuvants remains elusive. Due to increasing demand for novel adjuvants, a clearer understanding of the mechanisms that allow these important agents to affect adaptive immune responses will make a significant contribution to the rational design of future vaccines.

Using a novel approach to tracking antigen and antigen presentation, we demonstrate that alum induces higher antigen accumulation and increased antigen presentation by dendritic cells (DCs) *in vitro*. Antigen accumulation was 100-fold higher and antigen presentation 10-fold higher following alum treatment when compared with soluble protein alone. We also observed that alum causes an initial reduction in presentation compared with soluble antigen, but eventually increases the magnitude and duration of antigen presentation. This was associated with reduced protein degradation in DCs following alum treatment. These studies demonstrate the dynamic alterations in antigen processing and presentation induced by alum that underlie enhanced DC function in response to this adjuvant.

## Highlights

► The EαGFP/YAe system can be used to study the impact of alum on antigen uptake and presentation by DCs. ► Alum acts as an antigen delivery system. ► Alum slows down protein degradation in DCs. ► Alum eventually enhances magnitude and duration of expression of peptide/MHC complexes on the DC surface.

## 1 Introduction

Despite the tremendous variety of compounds with adjuvant activity, effective adjuvants for use in vaccines against major diseases such as Human Immunodeficiency Virus/Acquired Immunodeficiency Syndrome (HIV/AIDS), tuberculosis and malaria remain elusive [1]. A significant obstacle to the development of new and improved adjuvants is our lack of knowledge of their mechanism of action. This is particularly true of the aluminium adjuvants (alum) that have been applied in many vaccines since the 1920s [2]. It has been proposed that vaccine adjuvants act indirectly *via* DCs or other antigen presenting cells (APCs) to induce and enhance the activation of antigen-specific T cells and subsequently the adaptive immune response. CD4^+^ T cells play a central role in cell-mediated immunity and are activated in response to specific vaccine-derived, peptide epitopes bound to MHC class II (MHCII) molecules, displayed on the surface of APCs [3–5]. DCs possess highly controlled antigen processing functions utilising lysosomal proteases and pH changes optimal for the generation of peptides rather than complete protein degradation [6–8]. The duration and magnitude of antigen presentation are key factors in determining the degree and quality of T cell activation [9,10]. Although it has been suggested that an antigen pulse of a few hours is sufficient to support subsequent T cell division [11], the continued engagement of the peptide:MHCII:T cell receptor (p:MHCII:TCR) complex is required for a high degree of T cell expansion [12,13]. Adjuvants such as alum have been proposed to alter the magnitude and duration of antigen presentation through mechanisms such as increased antigen uptake [14,15] and enhanced expression of MHC class II molecules on the surface of APC [16–18]. While the impact of these factors on antigen presentation has previously been read out in terms of T cell expansion [15,19,20], these studies do not directly explain whether alum impacts on the magnitude and duration of antigen presentation that subserves adjuvant function.

To address this limitation, in the current study we have employed the chimaeric fluorescent protein, Ealpha green fluorescent protein (EαGFP), which allows assessment of antigen uptake/degradation and, in combination with the YAe antibody, antigen presentation *in situ* [21–24]. When EαGFP is internalised by DCs, it is degraded, liberating the Eα peptide for presentation on MHC class II molecules. This p:MHC complex can be detected by staining the cells with the monoclonal YAe antibody which specifically recognises the Eα:I:A^b^MHCII complex [21–24]. Using the EαGFP/YAe system, we have demonstrated that formulation of antigen in alum leads to an increase in antigen uptake, a decrease in antigen processing with the eventual result being enhanced magnitude and duration of antigen presentation by DCs *in vitro*.

## 2 Materials and methods

### 2.1 Preparation of DCs from murine bone marrow

Six- to eight-week-old C57BL/6 mice (H-2^b^) were used to prepare BMDCs, as described previously [25]. Mice were housed in the Central Research Facility, University of Glasgow and procedures were performed according to U.K. Home Office regulations. Bone marrow cells (2 × 10^6^/well) were placed in 6-well plates (Corning Incorporated, Corning, NY, USA) and cultured using 10% GMCSF (63× supernatants) supplemented with RPMI [RPMI 1640 (Sigma, UK), 10% FCS (Gibco, UK), 100 μg/mL penicillin and streptomycin (Invitrogen, UK) and 100 μg/mL l-glutamate (Invitrogen, UK)] at 37 °C in 5% CO_2_. At day 3 and 6, cells were fed with each 2 mL/well fresh complete DC media. DCs were used at day 7 for the experiments.

### 2.2 Antigens and adjuvants

The fluorescent antigen, EαGFP, was prepared in our laboratory using methods described previously [24]. Lipopolysaccharide (LPS; *Escherichia coli* O111.B4) was bought from Sigma and was used at 1 μg/mL concentration for positive control of MHC class II expression. ALHYDROGEL^®^ was bought from Brenntag Bioscience, Denmark. This adjuvant consists of 3% aluminium hydroxide.

### 2.3 Analysis of antigen uptake and presentation

To assess the role of alum in antigen uptake and presentation, we incubated BMDCs with different concentration of EαGFP or EαGFP adsorbed to different concentrations of alum in a 6-well plate containing 2 × 10^6^ cells/5 mL media in each well. Control wells were incubated with media.

To assess the role of alum in the kinetics of antigen uptake, degradation and presentation, we performed a pulse chase assay. BMDCs (3 × 10^6^) were pulsed with pre-determined doses of EαGFP or EαGFP adsorbed to alum for 1 h. Some of the cells were incubated in media for experimental control. Cells were harvested, washed in HBSS buffer (GIBCO, Invitrogen) (400 × *g*, 5 min, 4 °C) and BMDC separated from alum using sterile histopaque (Sigma cat no. H8889; 400 × *g*, 25 min, 20 °C) followed by washing twice in HBSS buffer (400 × *g*, 5 min, 4 °C). BMDC (1.5 × 10^6^ cells/5 mL) were resuspended in each well of a 6-well plate containing complete DC media and incubated for different chase periods (0 h, 24 h, 48 h and 72 h). After each chase period, cells were analysed by flow cytometry.

### 2.4 Flow cytometry

The cells were collected in 6 mL fluorescence-activated cell sorting (FACS) tubes (BD FALCON, BD Biosciences Discovery LabWare, USA) and washed (400 × *g*, 5 min, 4 °C) in FACS buffer (5% FCS, 0.1% sodium azide) and incubated with 100 μL Fc block (2.4G2 hybridoma supernatant) for 30 min. BMDCs were stained with phycoerythrin (PE) anti-mouse CD11c (eBiosciences, clone: N418, cat no. 12-0114), PE armenian hamster IgG (eBiosciences, clone: eBio299Arm, cat no. 12-4888-81), biotinalyted anti-mouse Eα52-68 (eBiosciences, clone: eBioYAe, cat no. 13-5741), bio anti-mouse IgG2b (Southern Biotech, clone: A-1, cat. 0104-08), bio Rat IgG2b, k (BD biosciences, cat no. 553987), allophycocyanin (APC) streptavidin (eBiosciences, cat no. 17-4317), peridinin chlorophyll protein (PerCP) anti-mouse I-A/I-E (BioLegends, clone: M5/114.15.2, cat no. 107623), PerCP streptavidin (BD Bioscience, cat no. 554064) for 30 min. Cells were washed twice (400 × *g*, 5 min, 4 °C) in FACS buffer and analysed by flow cytometry (BD Bioscience, FACS calibur). The results of flow cytometry were analysed by FlowJo software (FlowJo 8.7.1, Stanford University 1995–96). The level of GFP, YAe and MHC class II molecules present on CD11c positive cells were analysed by both mean fluorescence intensity (MFI) and percentage positive cells as determined using isotype controls.

### 2.5 Data analysis

Data were analysed using GraphPad Prism version 5.00 for Windows, GraphPad Software, San Diego, CA, USA. Results were expressed as mean ± SEM unless otherwise stated. In the data with one independent variable, we used Tukey post test (one way ANOVA) to test significance between any two different treatment groups. Similarly, in the data having more than one independent variable, we used Bonferroni post test (two way ANOVA) to test the significance between any two different treatment groups either at specific dose or at specific time. A *p*-value of < 0.05 was considered as significant.

## 3 Results

### 3.1 The EαGFP/YAe system can be used to study the impact of alum on antigen uptake and antigen presentation by bone marrow DCs (BMDCs)

**Fig. 1:**
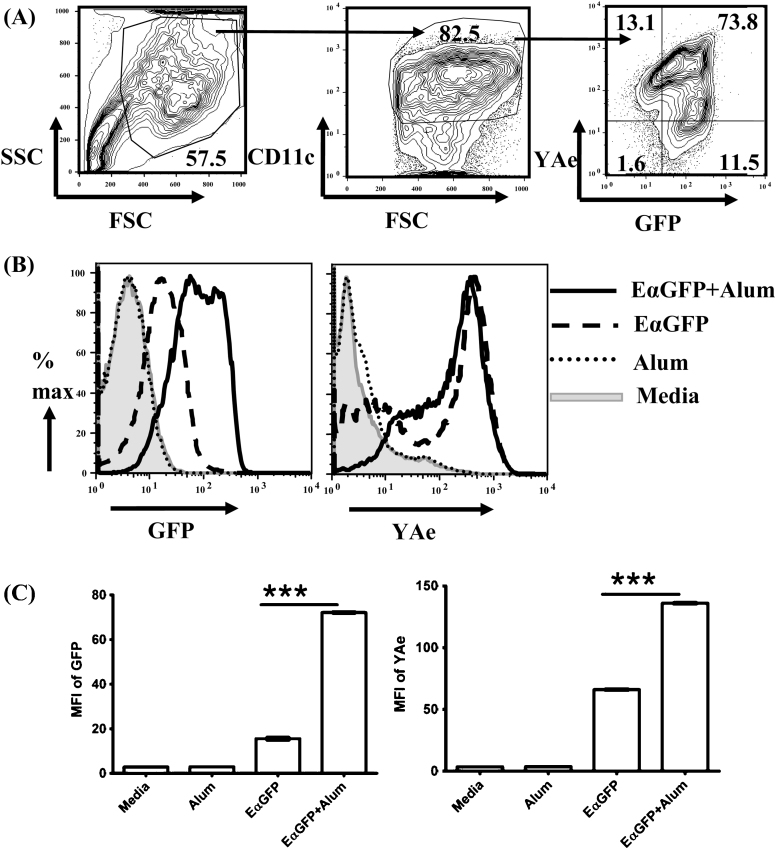
Application of the EαGFP/YAe system to reveal the impact of alum adjuvants on antigen uptake and presentation by DCs. BMDCs (2 × 10^6^/5 mL) were incubated in media, alum (100 μg/mL), EαGFP (100 μg/mL) and EαGFP adsorbed to alum (100 μg/mL) for 24 h. (A) A total of 50,000 cells were analysed on the basis of FSC (forward scatter) and SSC (side scatter) and DCs identified by CD11c expression. Analysis of GFP and YAe levels was performed on CD11c positive populations. These histograms represent the cells treated with EαGFP adsorbed to alum. (B) The overlay histograms represent the levels of GFP (left) and YAe (right) detected in DCs following different treatments as indicated in legends. (C) The bar graphs show the MFI of GFP (left) and MFI of YAe (right) in different treatment groups. Results are expressed as mean ± SEM of triplicate cultures. ***p < 0.0001 after comparing level (mean ± SEM) of GFP or YAe between EαGFP- and EαGFP + alum-treated groups as analysed by Tukey post test. Data shown is representative of five independent experiments.

To determine the suitability of the EαGFP/YAe system to investigate the impact of alum on antigen uptake and presentation, BMDCs were incubated with protein, alum-adsorbed protein or in the presence of media or alum alone for 24 h. We then analysed the level of GFP and YAe staining within the CD11c positive population (see Fig. 1A). Detection of GFP or YAe staining was dependent on the presence of EαGFP; incubating BMDC with alum alone did not produce any increase in either of these parameters compared with control cultures (see Fig. 1B and C). Adsorption of EαGFP to alum produced a significant increase in antigen uptake and presentation (*p* < 0.0001), with about 5-fold higher MFI of GFP and 2-fold higher MFI of YAe compared with cells incubated in EαGFP alone (see Fig. 1C). Similar results were obtained by analysing the proportion of GFP or YAe positive cells (data not shown). The results suggest that the EαGFP/YAe system is appropriate for the *in vitro* study of antigen uptake and presentation by DCs following alum treatment.

### 3.2 Alum acts as a delivery vehicle and targets antigen acquisition by DCs *in vitro*

**Fig. 2:**
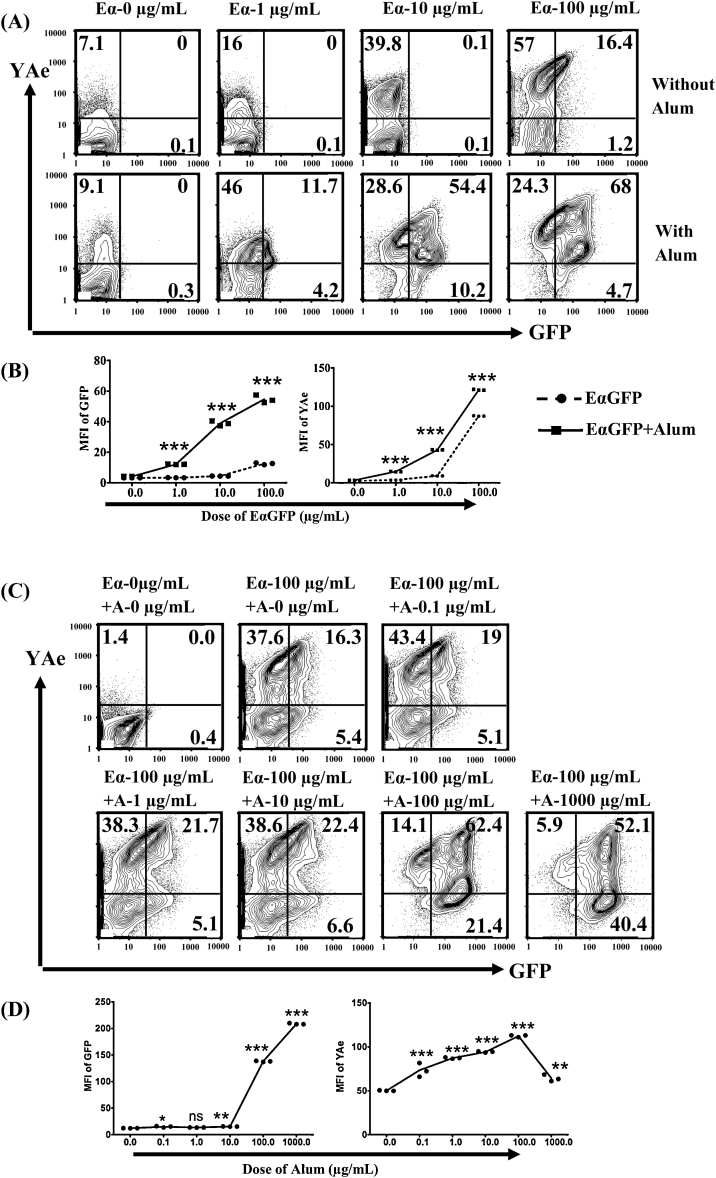
Alum efficiently targets DCs by enhancing both accumulation and presentation of antigen by DCs *in vitro*. (A) BMDCs (2 × 10^6^/5 mL) were incubated with Eα (EαGFP) (0, 1, 10, 100 μg/mL) and EαGFP adsorbed to alum (100 μg/mL) for 24 h and using the gating strategy in Fig. 1A, levels of GFP and or YAe on CD11c positive populations were analysed. (B) The levels of GFP (left) and YAe (right) on CD11c positive populations were analysed and the line graphs were made by the scatter dot plots and joining the mean values. Bonferronni post tests (two way ANOVA) were used to compare the level (mean ± SEM of triplicate cultures) of GFP or YAe between EαGFP- or EαGFP + alum-treated groups at particular dose of EαGFP. ***p < 0.001. Data shown is representative of three independent experiments. (C) BMDCs (2 × 10^6^/5 mL) were incubated with EαGFP (0 and 100 μg/mL) and EαGFP adsorbed to alum (0.0, 0.1, 1.0, 10.0, 100.0 and 1000.0 μg/mL) for 24 h and using the gating strategy in Fig. 1A, levels of GFP and/or YAe on CD11c positive populations were analysed. (D) The levels of GFP (left) and YAe (right) on CD11c positive populations were analysed and the line graphs were made by the scatter dot plots and joining the mean values. Tukey post test (one way ANOVA) was used to compare the level (mean ± SEM of triplicate cultures) of GFP or YAe between control (alum; 0.0 μg/mL or EαGFP; 100 μg/mL)-treated group and increasing concentrations of alum. ***p < 0.0001, **p < 0.001, *p < 0.01, ns = not significant. Data shown is representative of three independent experiments. Eα: EαGFP; A: alum.

Adsorption of EαGFP to alum significantly enhanced antigen uptake and presentation compared with soluble antigen over a range (1–100 μg/mL) of antigen doses tested (see Fig. 2A and B). Comparing the antigen dose response curves demonstrated that compared with soluble antigen, alum could induce similar antigen uptake by BMDC at a 100-fold lower dose (see Fig. 2B). Similarly, we observed the equivalent levels of YAe expression on cells incubated with 1 μg/mL EαGFP/alum compared with 10 μg/mL EαGFP alone suggesting that alum causes 10-fold increase in antigen presenting efficiency of DCs *in vitro* (see Fig. 2B). We next determined if the enhanced antigen uptake and presentation induced by alum was dependent on the dose of the adjuvant used. Using a fixed amount of EαGFP (100 μg/mL), doses of alum between 0.1 and 10 μg/mL produced a small, though significant increase in antigen uptake compared with soluble antigen (see Fig. 2C and D). This was reflected in a corresponding significant increase in presentation of the Eα peptide. Higher doses of alum (100 and 1000 μg/mL) produced much greater increase in GFP signal in BMDC, however the impact on antigen presentation was more modest, with decreased presentation being observed between 100 and 1000 μg/mL alum (see Fig. 2C and D). The difference in antigen presence and presentation in BMDC implied that the presence of alum may affect the rate of antigen degradation.

### 3.3 Alum adjuvants increase antigen uptake, reduce degradation and sustain antigen presentation by DCs

**Fig. 3:**
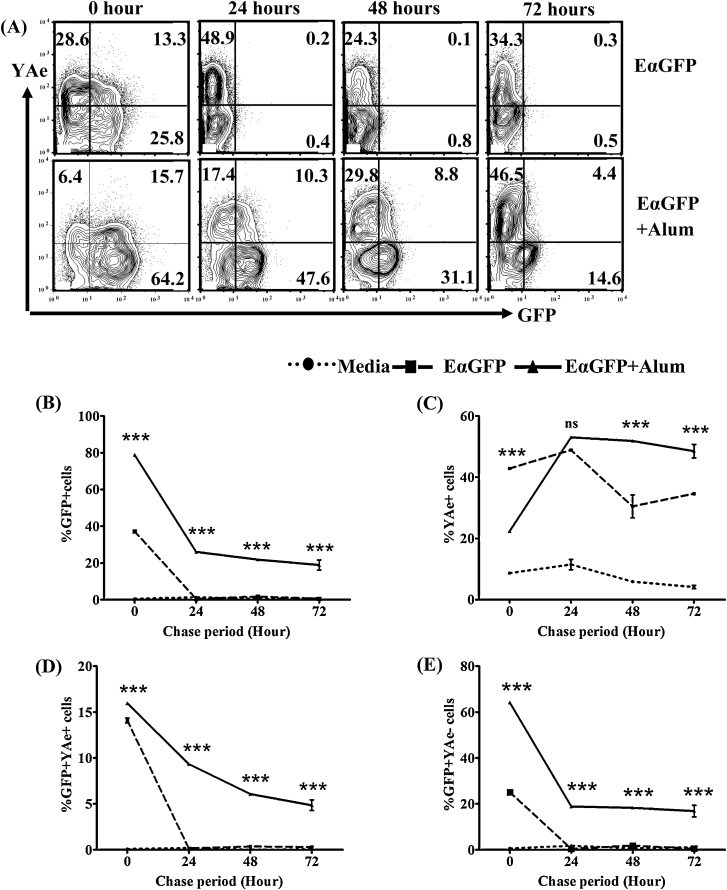
Alum enhances antigen uptake, reduces antigen degradation and maintains antigen presentation by DCs *in vitro*. (A) BMDCs (3 × 10^6^/5 mL) were pulsed with EαGFP (100 μg/mL) or EαGFP adsorbed to alum (100 μg/mL) for 1 h, then incubated for different chase periods (0, 24, 48 and 72 h). Using the gating strategy in Fig. 1A, levels of GFP and or YAe on CD11c positive populations were analysed. The line graphs show the % of GFP (B), % of YAe (C), % of GFP + YAe^+^ positive cells (D) and % of GFP + YAe^−^ (E) in different treatment groups. Data have been presented as the mean ± SEM of quadruplicate samples. Bonferronni post test (two way ANOVA) was used to evaluate *p*-value by comparing the proportion (mean ± SEM) of cells positive for either GFP and/or YAe between EαGFP- and EαGFP + alum-treated groups at specific chase period. ***p < 0.001, ns: not significant. Data shown is representative of two independent experiments.

To understand the role of alum in the kinetics of antigen uptake, processing and presentation by DCs we performed a pulse-chase experiment. We exposed BMDC to a 60 min pulse of antigen and examined antigen degradation and presentation over time. We found a greater proportion of cells were GFP positive (see Fig. 3A and B) and had a greater GFP signal, as determined by assessment of MFI (data not shown), following treatment with EαGFP adsorbed to alum compared with exposure to EαGFP at each chase period tested. Following exposure to soluble antigen, both the GFP signal and proportion of cells that were GFP positive returned to background levels within 24 h. The rate of GFP decay in cells exposed to antigen formulated in alum, was slower and the GFP signal was sustained up to 72 h following exposure (see Fig. 3B), demonstrating that intact antigen was degraded more slowly and persisted for longer in the presence of alum.

This would suggest that alum may also affect the rate of antigen presentation by BMDC. In keeping with this observation, formulation of antigen with alum decreased the fraction of YAe-positive BMDC at 0 h compared with soluble antigen (*p* < 0.01) which then gradually increased to an equal level as induced by EαGFP treatment at 24 h (*p* > 0.05) (see Fig. 3A and C). Alum significantly enhanced antigen presentation at 48 h (*p* < 0.001) which was sustained up to 72 h (*p* < 0.001) (see Fig. 3A and C). Similar trends were observed while analysing the data on the basis of MFI of the YAe (data not shown). We also examined the BMDC that were GFP positive for presence or absence of antigen presentation as detected by YAe staining. While the GFP + YAe^−^ population became undetectable within 24 h chase when pulsed with soluble EαGFP, these cells remained detectable for up to 72 h following alum/EαGFP treatment (see Fig. 3E). Very few cells were GFP + YAe^+^ following a 24 h chase period, this was more apparent in BMDC treated with soluble antigen than EαGFP adsorbed to alum (see Fig. 3D). This suggests that BMDC do not appear to transition through a GFP^+^/YAe^+^ population when moving from GFP positivity to YAe presentation and furthermore, that alum slows down antigen processing and presentation.

In summary, as well as targeting antigens to DC, data from these pulse chase experiments suggest that alum decreases the rate of antigen degradation resulting in increased duration and magnitude of antigen presentation by BMDC.

### 3.4 Alum increases MHC class II expression in DCs in dose-dependent manner

**Fig. 4:**
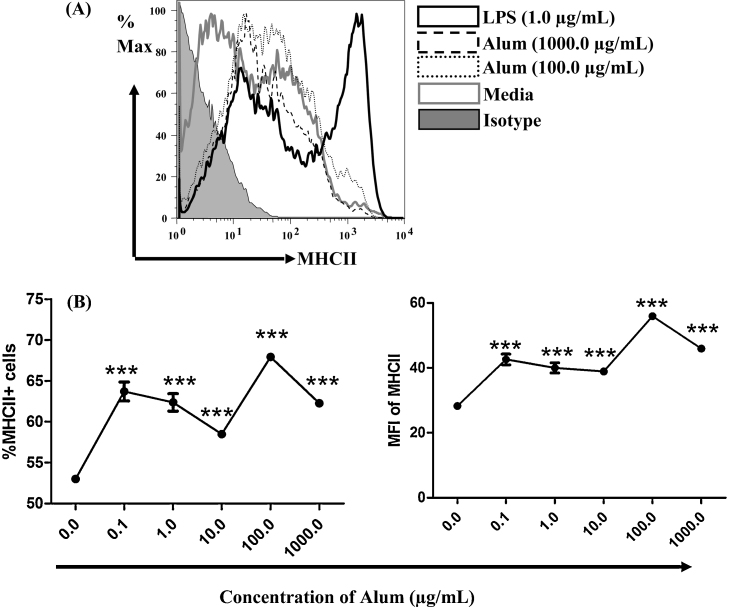
Alum increases level of MHC class II molecules in dose-dependent manner *in vitro*. BMDCs (2 × 10^6^/5 mL) were incubated with media, or with different doses of alum (0.1, 1, 10, 100 and 1000 μg/mL) and LPS (1 μg/mL) for 24 h. Cells were stained with anti-CD11c, anti-mouse MHC Class II and analysed by flow cytometry. (A) The overlay histograms show the % of maximum of cells positive for MHC class II molecules in BMDCs in different treatment groups. Shadow represents isotype control, which has been gated as negative for MHCII molecules. (B) The line graphs show the proportion (mean ± SEM) of MHCII positive cells (left) and the MFI (mean ± SEM) of MHC class II molecules (right) at different concentrations of alum in triplicate cultures. Tukey post tests (one way ANOVA) was used to compare the level of MHCII between untreated cells (incubated in media; alum-0.0 μg/mL) and cells treated with various concentrations of alum. ***p < 0.0001. Data shown is representative of two independent experiments.

While alum may act to make processed peptides available for longer, to mediate increased antigen presentation would presumably require increased cell surface MHC class II expression. We therefore analysed levels of MHC class II expression on BMDC incubated with different concentrations of alum (see Fig. 4A and B). We found that expression of MHC class II was dependent on the dose of alum used, with both the highest proportion of the cells positive for MHC class II molecules and the highest expression level of MHC class II molecules in the cells treated with 100 μg/mL of alum (see Fig. 4B). BMDC treated with 1000 μg/mL had lower MHC class II expression than the cells treated with 100 μg/mL alum, but higher than other doses used in the experiment indicating alum at high doses sustain MHC class II expression on the surface of DCs (see Fig. 4B). A similar dose response was seen for YAe expression in Fig. 2D, suggesting a link between increased antigen presentation and increased availability of MHC class II molecules following alum treatment *in vitro*.

## 4 Discussion

**Fig. 5:**
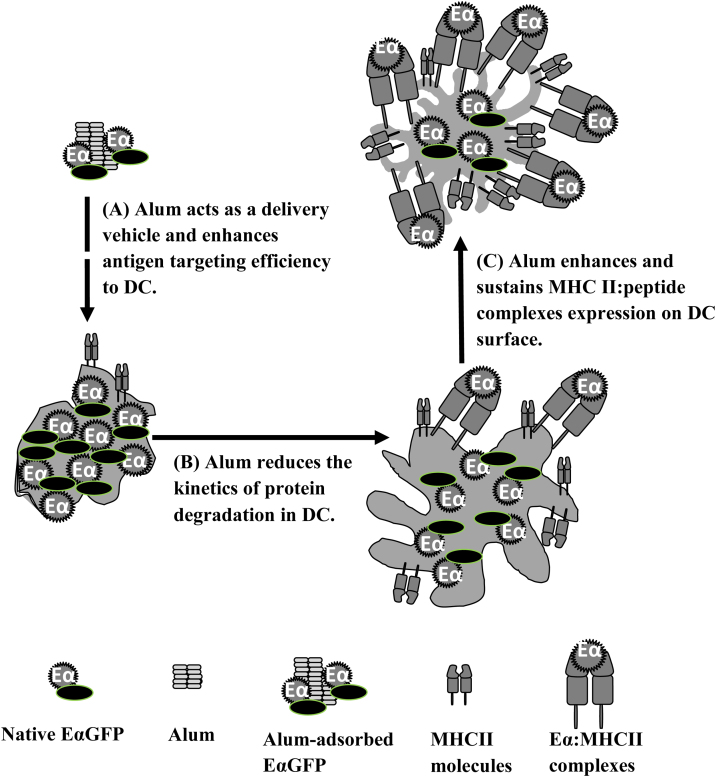
Illustrative conclusion of mechanisms of alum adjuvants *in vitro*. (A) Alum acts as an antigen delivery system. (B) Alum slows down protein degradation, presumably increasing the duration of peptide availability intracellularly. (C) Alum enhances magnitude and duration of expression of p:MHCII complexes on the DC surface.

In the current study we have applied the previously described EαGFP/YAe system [21,24] to directly track antigen internalisation, degradation and presentation in BMDC and the impact that alum adjuvants have on the magnitude and kinetics of these processes. The fluorescent protein moiety in the chimaeric EαGFP antigen allowed tracking of antigen uptake and degradation. This approach confirmed previous *in vitro* studies demonstrating the ability of alum to enhance internalisation of antigens by DCs [14,15], more importantly; we were also able to demonstrate that alum has a significant impact on the rate of degradation of antigen within DCs. While the GFP signal was completely extinguished within 24 h of administration of soluble antigen, formulation in alum allowed intact antigen to persist for up to 72 h. Degradation of antigens by lysosomal proteases is an essential step in liberating peptide antigens from proteins, and agents that interfere with this process, such as protease inhibitors or inhibitors of lysosomal acidificaiton have been shown to reduce antigen presentation [26,27]. This would suggest that slowing of antigen degradation by alum may result in poorer peptide loading and antigen presentation on MHC class II molecules. However, by virtue of the ability of the YAe antibody to directly recognise Eα:MHCII complexes, we were able to show that alum actually enhances the duration of antigen presentation by BMDC from less than 24 h, observed with soluble antigen, to at least 72 h. In agreement with our data, previous work has demonstrated that limiting the susceptibility of antigens to lysosomal proteolysis actually acts to increase antigen presentation and immunogenicity [28]. In terms of adjuvant activity *in vivo*, slowing down antigen degradation and increasing antigen persistence makes physiological sense. It takes hours for peripheral DCs to migrate to draining lymph nodes where naive cognate T cells are resident. Furthermore, functional interactions between DCs and T cells are thought to occur over the following 48 h or more [21] and blockade or interruption of this interaction is known to block the development of effective T cell responses [12,29,30]. The reduction in the rate of antigen degradation we observed could therefore lead to a temporal increase in availability of peptide for binding to MHCII in peptide loading compartments resulting in increased duration and magnitude of antigen presentation, as we also observed. However, it remains unclear at this point, exactly how this mechanism works. In the current study we also demonstrated that exposure to alum/EαGFP induces increased cell surface MHC class II expression on BMDC. Similarly, previous studies have shown the high expression of MHCII molecules following alum treatment in human peripheral blood mononuclear cells *in vitro* [16–18]. Interestingly, in the current study the dose response of alum induced MHC class II expression was similar to that observed when detecting Eα:MHCII complexes using the YAe antibody. Previous studies have demonstrated that inhibition of lysosomal proteases enhances the stability of p:MHCII complexes and leads to increased accumulation of MHCII complexes on the DC surface [31]. Therefore, if alum was to block lysosomal proteolysis, as suggested by the antigen persistence described above, this would explain the increased cell surface MHCII expression, although further studies would be required to validate this hypothesis. In summary, we have identified how alum modulates the following key steps leading to antigen presentation that could underpin adjuvant function (see Fig. 5). Firstly, formulation of antigen in alum results in increased antigen internalisation by BMDC *in vitro*, consistent with the hypothesis that alum acts as an antigen delivery system (Fig. 5A). Subsequently, we have demonstrated that alum slows protein degradation, presumably increasing the duration of peptide availability intracellularly (Fig. 5B). Finally, we have demonstrated that alum enhances magnitude and duration of expression of p:MHCII complexes on the DC surface, with an accompanying increase in MHCII expression (Fig. 5C). These consequences may underlie the generation of the long lasting T cell responses *in vivo*.

## Conflict of interest

The authors declare no financial or commercial conflict of interest.
